# LOW GLUCOSE ENHANCES THE CYTOPROTECTIVE EFFECT OF METFORMIN AGAINST DOXORUBICIN-INDUCED CYTOTOXICITY

**DOI:** 10.1093/geroni/igz038.3486

**Published:** 2019-11-08

**Authors:** Fathima Ameer, Xiaomin Zhang, Gohar Azhar, Yingni Che, Jeanne Wei

**Affiliations:** Donald W. Reynolds Institute on Aging, UAMS, Little Rock, Arkansas, United States

## Abstract

Metformin, an oral anti-diabetic drug, is currently being investigated for its anti-aging properties and has also been used as adjunct therapy in cancer. Cancer is a disease of aging. Type 2 diabetes is also prevalent in older adults. We wanted to test the hypothesis that metformin could protect normal cells during chemotherapy treatment under different glucose conditions. We used C2C12 myoblast cells to study cellular bioenergetics, variations in gene expressions, and biochemical alterations induced by metformin and pegylated liposomal doxorubicin (L-Doxo) under low glucose (2.7 mM or 50 mg/dL) and normal physiologic glucose (5.5 mM, or 100 mg/dL) conditions. Using confocal microscopy, we noted that treatment of C2C12 cells with 30 µg/mL L-Doxo under low glucose and normal physiologic glucose conditions induced cellular defects. Furthermore, L-Doxo treatment dysregulated the expression of mitochondrial fission and fusion genes, which may influence transformation of the network’s connectivity. L-Doxo treatment significantly reduced mitochondrial oxygen consumption rate (OCR) and extracellular acidification rate (ECAR). However, pre-treatment with 100 nM metformin provided protection against L-Doxo-induced damage and increased cell viability and ATP levels in cells even under low glucose conditions. Our data provide further evidence by which low dose metformin exerts protective effects against L-Doxo, a chemotherapeutic drug, under low glucose conditions. Metformin appears to act via AMPKα, Raptor, and SRF, and has significant cellular protective effects that may be useful in cancer and/or aging.

